# Effect of Hyaluronic Acid/Trehalose in Two Different Formulations on Signs and Symptoms in Patients with Moderate to Severe Dry Eye Disease

**DOI:** 10.1155/2018/4691417

**Published:** 2018-08-01

**Authors:** Klemens Fondi, Piotr A. Wozniak, Doreen Schmidl, Ahmed M. Bata, Katarzyna J. Witkowska, Alina Popa-Cherecheanu, Leopold Schmetterer, Gerhard Garhöfer

**Affiliations:** ^1^Department of Clinical Pharmacology, Medical University of Vienna, Vienna, Austria; ^2^Department of Ophthalmology, Medical University of Warsaw, Warsaw, Poland; ^3^Carol Davila University of Medicine and Pharmacy, Bucharest, Romania; ^4^Department of Ophthalmology, Emergency University Hospital, Bucharest, Romania; ^5^Center for Medical Physics and Biomedical Engineering, Medical University of Vienna, Vienna, Austria; ^6^Singapore Eye Research Institute, Singapore National Eye Centre, Singapore; ^7^Lee Kong Chian School of Medicine, Nanyang Technological University, 59 Nanyang Drive, Singapore 637459; ^8^Visual Science Academic Clinical Programme, Duke-NUS, Singapore

## Abstract

**Purpose:**

This randomized, observer-masked, crossover study investigated the effect of two hyaluronic acid/trehalose-based containing formulations, with different physical properties, on the signs and symptoms in patients with moderate to severe dry eye disease (DED).

**Methods:**

In one group, patients received a mixture of sodium hyaluronate and trehalose (HT, Thealoz Duo®) for use during the day. In the other group, patients received a more viscous formulation consisting of hyaluronic acid, trehalose, and carbomer (HTC-gel, Thealoz Duo Gel) to use pro re nata. Both groups used HTC-gel before going to bed. Clinical standard tests for DED were performed at the beginning and end of each one-week period. Further, patient satisfaction including quality of sleep was assessed using a visual analogue scale.

**Results:**

Corneal fluorescein and conjunctival lissamine green staining scores decreased, and tear breakup time (BUT) increased for both groups (*p* < 0.001 each). Mean instillation frequency was 3.1 ± 2.6 drops/day when using HT and 1.9 ± 2.2 drops/day when using HTC-gel (*p*=0.02). A significant improvement in the quality of sleep was observed with both treatments (*p*=0.01).

**Conclusions:**

Our results show improvement in signs and symptoms of DED in both groups. While instillation of HTC-gel resulted in a lower instillation frequency, both formulations of trehalose showed good clinical efficacy. This trial is registered with NCT02980913.

## 1. Introduction

Dry eye disease (DED) is a frequent ocular disorder occurring especially in the elderly [1]. DED affects both the tears and the ocular surface and may produce symptoms that range from slight ocular discomfort up to a considerable reduction of quality of life caused by corneal damage, decreased vision, and pain [2, 3]. Although in recent years important progress has been made in the understanding of DED and its pathogenesis, tear supplementation is still a mainstay of therapy for DED.

Most tear supplements function as topical lubricants and are therefore designed to moisten the ocular surface. This is achieved by complex formulations, including a large variety of surfactants, emulsifiers, and electrolytes, which differ considerably between products [4–6]. Recently, trehalose, a potent osmo- and bioprotectant [6, 7], and castor oil [8, 9] have been introduced to tear supplements to exert biological effects beyond lubrication.

One major determinant of clinical efficacy, along with patient compliance, is the residency time of the topical lubricant therapy on the ocular surface. A short ocular residency time requires frequent instillation of the product [7, 10], which in turn limits patient compliance and quality of life. In principle, this can be overcome by increasing the viscosity of the lubricant formulation. This has been confirmed by a variety of studies showing that higher viscosity of the formulation is related to a longer ocular residence time [11–13]. Although this may lead to a better lubrication of the ocular surface, administration of gel-based topical lubricants is not well tolerated by all patients, because it may impact quality of vision. Thus, some treatment regimens use gels mainly as an evening therapy. Evidence from clinical studies regarding the effect of different administration schemes for high-viscous eye drops is, however, sparse.

Although a large number of different topical lubricants are currently approved and used in clinical routine, not much information is available on the clinical effect of different treatment schemes according to the formulation. The current study was designed to investigate the clinical effect of a new topical treatment with a lachrymal substitute gel, containing trehalose, hyaluronic acid, and carbomer (Thealoz Duo Gel, HTC-gel), compared to the use of artificial tears containing trehalose and hyaluronic acid (Thealoz Duo, HT) during the day. Both groups used the gel formulation before going to sleep. As a secondary aim, the effect of therapy on sleep-related parameters, daily work, and impact on daily activities was assessed. Further, the voluntary instillation frequency of the two different treatment schemes was also recorded.

## 2. Materials and Methods

### 2.1. Subjects

The study was performed in accordance with the Declaration of Helsinki and the Good Clinical Practice (GCP) guidelines of the European Union. The study protocol was approved by the Ethics Committee of the Medical University of Vienna. Forty male and female patients with moderate to severe DED were included according to the inclusion/exclusion criteria outlined below. All subjects gave written informed consent after detailed personal explanation.

Participating subjects underwent a prestudy screening, which was carried out within the 2 weeks before the first study day. The screening examination included a medical history for general and ocular conditions (including duration of DED), pregnancy test in women with childbearing potential, subjective assessment of symptoms of DED using the Ocular Surface Disease Index (OSDI) questionnaire, ophthalmic examination including visual acuity using the ETDRS acuity charts, slit-lamp biomicroscopy and indirect funduscopy, measurement of breakup time (BUT), Schirmer I test, and measurement of intraocular pressure (IOP).

Patients with moderate to severe dry eye disease were included based on the following criteria: OSDI ≥ 22 and BUT ≤ 10 s or Schirmer I test between 2 mm and 5 mm. Further inclusion criteria were age of at least 18 years, normal ophthalmic findings except DED, ametropia <6 diopters, and a history of DED for at least 3 months. The eye with the lower BUT was chosen as the study eye. If BUT was identical for both eyes, the eye with the lower Schirmer I test was chosen. If Schirmer I test results were also identical for both eyes, the right eye was used for study purposes.

The following exclusion criteria were defined for the present study: treatment with any ophthalmic drug except topical lubricants, intake of systemic NSAIDS, immunosuppressants, corticosteroids, or any medication which could affect lachrymal function (e.g., antidepressants, anxiolytics, neuroleptics, antihistamines, cholinergics, antimuscarines, phenothiazin, and beta-blockers). Further exclusion criteria were ocular surgery in the month preceding the study, known hypersensitivity to one of the administered products, pregnancy, planned pregnancy or lactating, participation in a clinical trial in the four weeks preceding the study, symptoms of a clinically relevant illness in the two weeks before the first study day, presence or a history of a severe medical condition as judged by the clinical investigator, and wearing of contact lenses.

### 2.2. Treatment Regimen

The following treatments were compared: one group received sodium hyaluronate/trehalose-based eye drops (HT, Thealoz Duo Eye Drops; Laboratoires Thea, France) instilled pro re nata (PRN) during the day for one week. The second treatment group received a sodium hyaluronate/trehalose/carbomer gel (HTC-gel, Thealoz Duo Gel, Laboratoires Thea, France) again instilled PRN for one week. Both treatment groups were asked to instill the HTC-gel at night time before going to sleep. After the first treatment period, the patients crossed over to the alternative treatment.

### 2.3. Study Design and Experimental Paradigm

**Figure 1:**
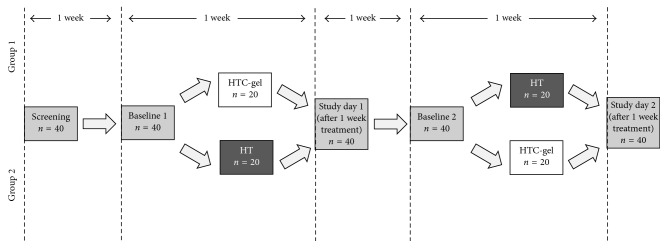
Randomization process and study design.

The present study was performed in a randomized, observer-masked two-way crossover design. Forty patients with moderate to severe DED who matched the in/exclusion criteria described above were included in the study. A schematic drawing explaining the study design is given in Figure 1.

Before the first randomization period, a 7-day washout period was scheduled. During this period, patients were handed out eye drops containing hyaluronic acid (Hyabak®, Laboratoires Thea, France) to use until three days before the start of the study. Thereafter, patients used saline eye drops (Hydrabak, Laboratoires Thea, France) until the first study day. Patients were asked to stop all topical lubricants 12 hours before the first study day. In addition, patients were handed out a diary to document instillation frequencies and times.

After the 7-day washout period, patients were randomized to receive either HT or HTC-gel for one week. Both groups used HTC-gel before going to sleep. After another washout period for 1 week, patients crossed over to the alternative treatment for one week.

During the study, patients were instructed to record instillation frequencies in a diary. In addition, clinical standard tests for DED, such as measurement of breakup time (BUT), corneal fluorescein, and conjunctival lissamine green staining, Schirmer I test, and completion of the Ocular Surface Disease Index (OSDI) questionnaire were performed at the beginning and end of each crossover period.

## 3. Methods

### 3.1. Breakup Time

Tear breakup time was measured following the guidelines described in the report of the TFOS International Dry Eye Workshop [14]. Five microliters of Minims-Fluorescein Sodium 2.0% eye drops was applied in the conjunctival sac of the eye, and the patients were instructed to blink naturally to distribute the fluorescein. The patients were asked to stare straight ahead without blinking, until told otherwise. By means of a stopwatch, the time between last complete blink and first appearance of a dry spot was recorded. The slit-lamp magnification was set at 10x, the background illumination intensity was kept constant (cobalt blue light), and a yellow filter was used to enhance observation of the tear film over the entire cornea. The mean value of three consecutive measurements was used for analysis.

### 3.2. Corneal Fluorescein Staining

**Figure 2:**
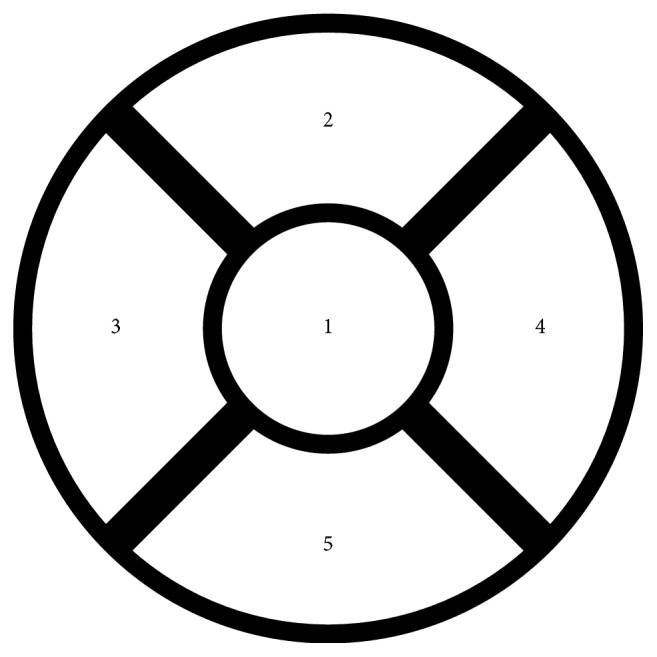
Divisions of the cornea according to the NEI/Industry Workshop guidelines [15].

Minims-Fluorescein Sodium 2.0% eye drops were used to detect corneal epithelial defects using cobalt blue light and a yellow filter. As a grading scale for corneal damage, the NEI/Industry Workshop guidelines were used [15]. The cornea was divided into five sectors (central, superior, inferior, nasal, and temporal), each of which was scored on a scale of 0–3, with 0 meaning no staining and 3 meaning maximum staining, with a maximal overall score of 15 (Figure 2).

### 3.3. Conjunctival Staining with Lissamine Green

Lissamine green (LG, EasyOpht, Italy) was used to detect conjunctival defects. Before placing the strip in the lower fornix of the eye, a drop of sterile saline was added to the strip. As grading scale for conjunctival damage, the NEI/Industry Workshop guidelines were used [15]. Both nasally and temporally, the conjunctiva was divided into a superior paralimbal area, an inferior paralimbal area, and a peripheral area with a grading scale of 0–3, with 0 meaning no staining and 3 meaning maximum staining, with a maximal score of 9 for the nasal and temporal conjunctiva, respectively.

### 3.4. Schirmer I Test

Schirmer I test without anesthesia was performed following the guidelines published in the report of the TFOS International Dry Eye Workshop [14]. Schirmer paper strips were inserted in the eye over the lower lid margin, midway between the middle and outer third. The patient was asked to close the eye, and after 5 minutes, the wetting of the Schirmer paper was measured.

### 3.5. Intraocular Pressure

Intraocular pressure (IOP) was measured with a slit-lamp mounted Goldmann applanation tonometer. Before each measurement, one drop of oxybuprocaine hydrochloride (4.0 mg/mL) combined with sodium fluorescein (0.8 mg/mL) was used for local anesthesia.

### 3.6. Ocular Surface Disease Index (OSDI)

Symptoms of dry eye were assessed using the OSDI. The questionnaire underlying the OSDI is specifically designed for patients with DED and asks patients about the frequency of specific symptoms and their impact on vision-related functioning. Results are reported on a scale from 0 to 100, whereas “0” means no symptoms of DED.

### 3.7. Patient's Satisfaction and Questionnaires

Patient satisfaction scores were determined using a 100 mm VAS on which 0 means no symptoms and 100 means the worst possible discomfort. Patients were asked to mark the 100 mm line at the corresponding value on the VAS. The following parameters were assessed using VAS: patient satisfaction when awakening, feeling of swollen eyelid when awakening, ocular comfort during the night, and quality of sleep.

The effect of therapy on daily work and impact on daily activities was assessed using a 4-grade scale (0 = absent, 1 = present but not disturbing, 2 = disturbing but not interfering with daily life, and 3 = very distressing and interfering with daily activities).

### 3.8. Data Analysis

Statistical analysis was done “per protocol.” To detect differences between the two treatment groups, a repeated measures ANOVA model was applied. Planned comparisons within the ANOVA model were performed to assess differences in clinical signs, subjective parameters, and instillation frequency between time points and groups. A *p* level of 0.05 or less was considered significant. Baseline values were reported using descriptive results (frequencies and percentages or mean and standard deviation). To detect differences in variables that were based on a 4-point scale, Kruskal–Wallis ANOVA was used. All statistical analyses were carried out using CSS Statistica (Version 6.0, Tulsa, Oklahoma, US).

## 4. Results

A total of 45 patients with dry eye disease aged between 20 and 72 years were included, of which 40 completed the study according to the protocol (age: 43.7 ± 12.3 years, mean ± SD). Of those participants, 12 patients were male and 28 patients were female. Two patients had to be excluded because they took prohibited medication, 1 patient withdrew consent and 2 patients were lost to follow up. The mean duration of DED in the whole study group was 6.9 ± 6.1 years. Mean OSDI score at screening was 51.8 ± 15.5.

After one week of treatment, BUT significantly increased and corneal fluorescein staining score significantly decreased (*p* < 0.001 each) in both study groups. In addition, a significant decrease in lissamine green staining scores was observed in both groups after one week of treatment (*p* < 0.001). In both groups, OSDI tended to decrease after one week of treatment (*p*=0.06). No significant changes in Schirmer I test scores were observed (*p*=0.88). Absolute values for these parameters are shown in Table 1.

**Figure 3:**
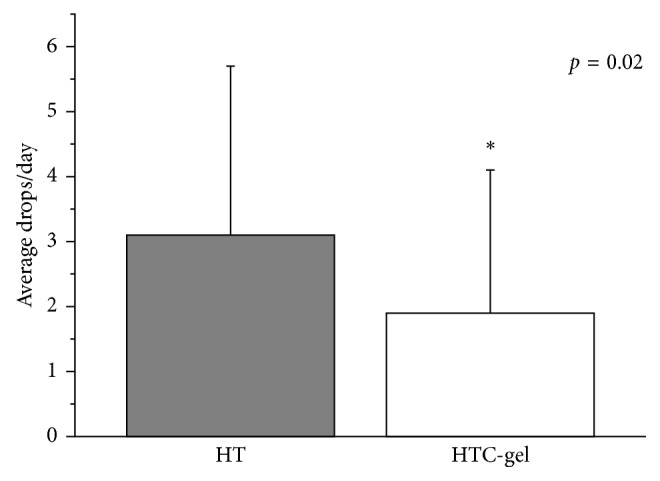
Difference in the frequency of use when using a gel containing hyaluronic acid, trehalose, and carbomer (HTC-gel) PRN compared to using eye drops containing hyaluronic acid and trehalose (HT) PRN.

During HT treatment, the frequency of instillation was 3.2 ± 2.6 times per day. When treated with HTC-gel, the frequency of instillation was significantly lower (1.9 ± 2.2 times per day, *p*=0.02) compared to the HT treatment (Figure 3).

**Figure 4:**
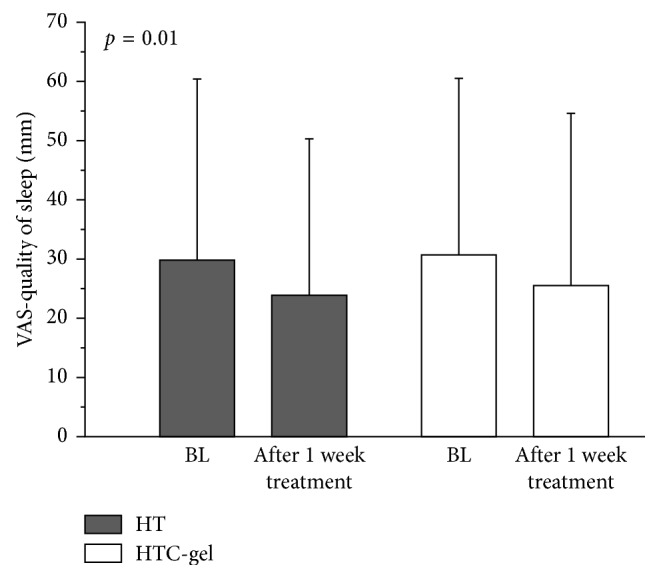
Patients' quality of sleep on the VAS at baseline and after one-week treatment with a gel containing hyaluronic acid, trehalose, and carbomer (HTC-gel) PRN compared to using eye drops containing hyaluronic acid and trehalose (HT) PRN.

When looking at the VAS scores, a significant improvement in the quality of sleep was observed with both treatment regimens (*p*=0.01, Table 2, Figure 4). For the other parameters evaluated, no significant changes occurred, although a tendency towards improvement in mean VAS scores for all parameters taken together was observed (Table 2).

The Kruskal–Wallis ANOVA revealed no differences in terms of changes of the impact on work and daily activities between the two study groups. This was also true after one week of treatment in either of the two groups (data not shown). No changes in IOP or visual acuity were observed during the course of the study (data not shown).

## 5. Discussion

The data of the current study indicate that treatment with Thealoz Duo Gel alone or with Thealoz Duo eye drops combined with the gel before going to bed, led to a comparable improvement in BUT, corneal fluorescein, and conjunctival lissamine green staining scores. Both treatment regimens also significantly improved patients' quality of sleep. Further, the use of eye drops with higher viscosity during daytime was associated with a lower instillation frequency compared to the combination of the less viscous formulation during the day and the lubricant gel at night.

Modern tear replacement therapies are usually based on complex formulations consisting of different chemical components such as cellulose derivates, polyacrylic acid, or hyaluronic acid. Although the latter components exert good lubricant effects, new topical DED therapies also include agents that exert effects beyond wetting of the ocular surface, in particular, small organic molecules such as trehalose, L-carnitine, or some polyols such as erythritol [6, 16]. There is increasing evidence now that these osmoprotectants directly protect cells against hyperosmolarity, which is one of the main causes of cell death and inflammation in DED [6].

However, despite many efforts, there is currently no gold standard for the topical therapy for DED. A common problem and an important limitation in the treatment with topical lubricants is that the association between clinical effects and different treatment schemes is only insufficiently described [7, 10]. In particular, data from clinical trials regarding the influence of viscosity on instillation frequency and clinical efficacy are sparse.

The data of the current study indicate that both treatment regimens led to a comparable improvement in clinical signs of DED. It is worth noting that this improvement in clinical variables was observed even after a short-time treatment of one week. In particular, BUT, corneal fluorescein, and conjunctival lissamine green staining scores were significantly improved in both groups to a similar degree. This indicates that a strong beneficial effect on clinical signs is present with both treatment regimens, and that the treatment scheme may be adapted to the patients' preference.

A significant improvement in patient's quality of sleep was observed with both treatment regimens. This is of special importance since several studies have reported that poor sleep quality is associated with DED and the severity of symptoms [17, 18]. Further, patients' symptoms, as measured in this study using the OSDI score, showed a tendency towards improvements in both groups, but this effect did not reach the level of significance. Again, no significant difference was observed between the two treatment groups, indicating that although the HTC-gel treatment requires less frequent instillation, the patient-related benefit seems to be comparable.

Furthermore, our results show that the instillation frequency is significantly lower when patients were using HTC-gel during the day in comparison to the less viscous HT formulation. It is reasonable to suggest that the reason for this finding is related to the physical properties of the two formulations under the study. The HTC-gel used in the current trial contains a carbomer component in addition to hyaluronic acid/trehalose, which are the main components of the HT formulation. It has been hypothesized that carbomer comprising gels significantly extend the contact time of solutes or suspended solids with the corneal surface [19]. Therefore, it can be expected that using a HTC-gel formulation will lead to a less frequent instillation frequency, which is also reflected in the data of the current study.

The physical properties of the HT combination may well explain the lack of clinical difference between the two formulations, although the treatment frequency was higher in the HT group. Although the HT formulation does not contain a high-viscous component, it shows a long residency time on the ocular surface. In particular, it has been shown in a previous study that eye drops containing trehalose and hyaluronic acid, that were also used in the present study, increase tear film thickness as measured with a ultrahigh resolution optical coherence tomography system for as long as 240 minutes, indicating long corneal residence time [20].

In the current study, we have used a two-way crossover design, which is usually considered the gold standard in clinical trials. However, one prerequisite for this study design is that patients start on the same severity level at the beginning of both treatment periods and that no overhang effect of the treatment is present. This was achieved by a 7-day washout period, which was scheduled before start of each treatment period. Furthermore, the washout allows the study to detect effects largely independent from the previous treatments, which is also been previously described as a beneficial way to improve the clinical trial design in DED [21]. It has, however, to be noted that a washout period with saline may, in principle, influence tear film stability and impact the ocular surface. To minimize this effect, only in the last 3 of 7 days, saline solution was used whereas patients received hyaluronic acid for the other days.

Further, no active control or placebo group was included in the study. However, one of the strengths of a crossover design is that each subject receives all treatments and therefore acts as its own control, which considerably reduces confounding covariates. Given that the main aim of this study was to compare the two different treatment regimens, we do not assume that it limits the conclusions of the study.

Finally, our study indicates that the safety profile of both treatment regimens was excellent. No change in any of the safety parameters was observed, and adverse events that occurred during the course of the study were mild. This is also reflected in the VAS scores of patients' satisfaction that showed a trend towards improvement.

Some limitations need to be considered when interpreting the results of our study. First, for feasibility reasons, the treatment time was limited to one week before patients crossed over to the alternative treatment. Thus, it cannot be fully excluded that the limited observation period was not sufficient to detect differences in clinical parameters between the two treatment regimens. This is also reflected in the clinical parameters, such as staining and BUT. Although both variables show a statistical significant improvement, longer studies are needed to assess the full clinical effect of the treatments.

Furthermore, the sample size of the current study was limited to 40 patients. Although the crossover design used in the current study is considered to be the gold standard for clinical studies because every patient acts as his/her own control, which provides additional power in comparison to parallel group studies, a larger sample size with longer observation periods may be necessary to detect subtle differences in signs and symptoms.

## 6. Conclusions

In conclusion, the data of the current study showed that both formulations containing trehalose and hyaluronic acid induced a significant improvement in BUT, corneal fluorescein, and conjunctival lissamine green staining scores and patient's quality of sleep after 1 week of use. Further, although the instillation frequency was lower when using the HTC-gel formulation compared to the more liquid HT eye drops, no statistical difference was found between the two treatment regimens regarding the improvement of clinical measures and symptoms of DED. Therefore, both trehalose-based treatment schemes are effective in patients with DED and can be adapted according to patient's preferences regarding the formulation.

## Figures and Tables

**Table 1:** Changes in clinical signs and symptoms after 1 week treatment with either a gel containing hyaluronic acid, trehalose, and carbomer (HTC-gel) or eye drops containing hyaluronic acid (HT) PRN.

	HTC-gel		HT		*p* value (time effect, ANOVA)
	Baseline	After 1-week treatment	Baseline	After 1-week treatment	
Breakup time (s)	3.5 ± 1.7	4.2 ± 1.7	3.4 ± 1.4	4.3 ± 1.9	<0.001
Corneal fluorescein staining (0–15)	3.5 ± 2.3	2.7 ± 2.1	3.1 ± 1.7	2.2 ± 1.4	<0.001
Conjunctival lissamine green staining (0–18)	4.5 ± 2.8	3.5 ± 2.6	4.4 ± 2.5	2.8 ± 2.3	<0.001
Schirmer test I (mm)	11.6 ± 9.6	11.0 ± 9.2	12.2 ± 10.6	13.0 ± 10.4	0.88
Ocular surface disease index (OSDI, 0–100)	37.3 ± 21.4	36.1 ± 21.5	37.3 ± 19.9	33.4 ± 20.2	0.06

**Table 2:** Changes in the visual analogue scale (VAS) for each question after 1 week treatment with either a gel containing hyaluronic acid, trehalose, and carbomer (HTC-gel) or eye drops containing hyaluronic acid (HT) PRN.

	Baseline	Study day	*p* value (time effect, ANOVA)	*p* value (between groups)
“What is your global satisfaction concerning your eyes when awakening in the morning?”				
HT	26.9 ± 22.5	25.0 ± 22.8	0.48	0.59
HTC-gel	27.1 ± 26.0	26.8 ± 25.1		

Feeling of swollen eyelid when awakening				
HT	23.9 ± 23.8	21.9 ± 24.5	0.68	0.38
HTC-gel	24.9 ± 25.5	25.6 ± 25.2		

Subjective evaluation of ocular comfort during the night				
HT	25.7 ± 23.1	26.2 ± 24.2	0.56	0.36
HTC-gel	27.0 ± 25.0	24.9 ± 23.3		

Quality of sleep				
HT	29.8 ± 30.6	23.9 ± 26.4	**0.01**	0.87
HTC-gel	30.7 ± 29.8	25.5 ± 29.1		

Mean VAS score				
HT	26.7 ± 21.3	23.7 ± 20.7	0.08	0.67
HTC-gel	27.5 ± 23.6	25.7 ± 22.5		

## Data Availability

The study was sponsored by Laboratories Thea and so the data cannot be made freely available. Access to these data will be considered by the corresponding author upon request, with permission of Laboratories Thea.
